# Supplementary material for: Effectiveness and Safety of Ayurvedic Medicines in Type 2 Diabetes Mellitus Management: A Systematic Review and Meta-Analysis
Source: Front Pharmacol. 2022 Jun 8;13:821810. doi: 10.3389/fphar.2022.821810 (PMC9213670; doi:10.3389/fphar.2022.821810)
Supplement: Supplementary file 1 [file DataSheet1.zip › Supplementary Material/Forest and Funnel Plots/Trigonella foenum-graecum/PPBG.pdf]

Trigonella foenum-graecum - PPBG

Study	Intervention N	Intervention Mean	Intervention SD	Comparator N	Comparator Mean	Comparator SD	Mean Diff. with 95% CI	Weight (%)
Gupta 2001	12	181.10	69.00	13	241.60	43.00	-60.50 [-105.17, -15.83]	6.74
Lu 2008	46	170.43	39.09	23	216.37	72.42	-45.94 [-72.18, -19.70]	12.67
Yaheya 2009	40	152.50	21.27	40	153.70	9.22	-1.20 [-8.38, 5.98]	22.15
Kaur 2016	30	183.10	12.26	30	200.27	11.70	-17.17 [-23.23, -11.11]	22.54
Verma 2016	77	199.38	70.57	77	226.31	77.97	-26.93 [-50.42, -3.44]	13.95
Kandhare 2018	59	232.46	76.04	53	254.49	84.93	-22.03 [-51.84, 7.78]	11.17
Hota 2019	102	153.73	103.81	102	156.60	119.99	-2.87 [-33.66, 27.92]	10.79
**Overall**							-20.56 [-34.29, -6.83]	

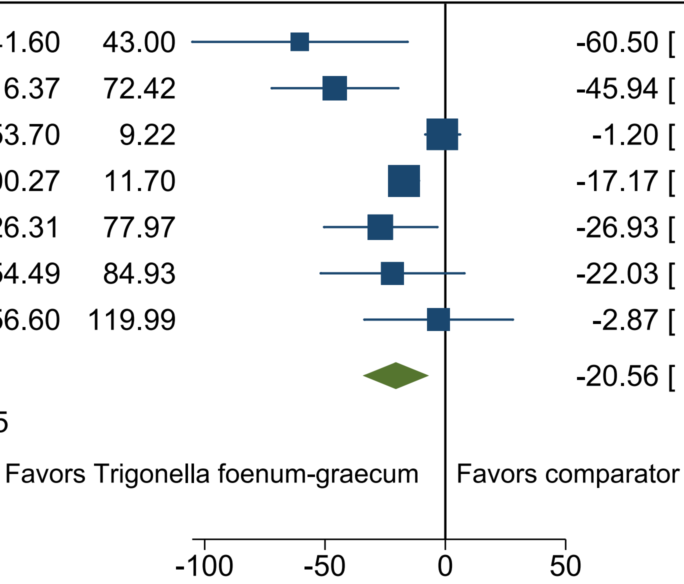

Heterogeneity: $\tau^2 = 208.09$, $I^2 = 80.57\%$, $H^2 = 5.15$

Test of $\theta_i = \theta_j$: Q(6) = 24.67, p = 0.00

Test of $\theta = 0$: z = -2.94, p = 0.00

Random-effects REML model
